# QM-DLA: an efficient qubit mapping method based on dynamic look-ahead strategy

**DOI:** 10.1038/s41598-024-64061-0

**Published:** 2024-06-07

**Authors:** Hui Liu, Bingjie Zhang, Yu Zhu, Hanxiao Yang, Bo Zhao

**Affiliations:** 1 College of Computer and Information Engineering, Henan Normal University, Xinxiang, 453007 Henan China; 2 Engineering Technology Research Center for Computing Intelligence and Data Mining in Henan Province, Henan Normal University, Xinxiang, 453007 Henan China; 3 Laboratory for Advanced Computing and Intelligence Engineering, Zhengzhou, 450001 China; 4 Information Engineering University, Zhengzhou, 450001 China

**Keywords:** Quantum computing, Qubit mapping, Quantum circuit, Dynamic look-ahead, Computer science, Quantum information

## Abstract

Quantum computing has already demonstrated great computational potential across multiple domains and has received more and more attention. However, due to the connectivity limitations of Noisy Intermediate-Scale Quantum (NISQ) devices, most of the quantum algorithms cannot be directly executed without the help of inserting SWAP gates. Nevertheless, more SWAP gates lead to a longer execution time and, inevitably, lower fidelity of the algorithm. To this end, this paper proposes an optimized qubit mapping algorithm based on a dynamic look-ahead strategy to minimize the number of SWAP gates inserted. Firstly, a heuristic algorithm is proposed based on maximizing physical qubit connectivity to generate the optimal initial qubit mapping, which reduces the need for logical qubit shifts during subsequent SWAP gate insertion. Secondly, in the form of directed acyclic graphs, we identify quantum gates that violate the constraints of physical coupling and insert SWAP gates to remap qubits, thereby overcoming the limitations of qubit interactions. Finally, the optimal SWAP gate insertion strategy is built by comparing the cost of different SWAP gate insertion strategies through a multi-window look-ahead strategy to reduce the number of SWAP gates inserted. The experimental results show that the strategy in this paper decreases the number of SWAP gate insertions and significantly reduces the depth of quantum circuits when performing qubit mapping compared with state-of-the-art methods.

## Introduction

Quantum computing, owing to its inherent attributes of quantum superposition and quantum entanglement, has demonstrated significant potential in numerous domains, including rapid data searching and sorting^1^, quantum chemistry^2^, machine learning^3,4^ and cryptography^5^, etc. Taking the factorization of large numbers as an example^6^, the most powerful supercomputing, Frontier, may need hundreds of million years to decompose a 2048-bit large number, while a general quantum computer is supposed to accomplish the same task in a few seconds merely. The lowest resource (qubits) estimate for the 2048 bit integer factorization to date is 3 million ion-trap qubits^7^.

With the release of 127, 49, and 72 qubit quantum devices by IBM, Intel, and Google, respectively, the NISQ era, a new era of quantum technology development, has come, indicating a crucial advancement toward the future’s more potent and powerful quantum technologies^8^. Unfortunately, the existing limitations of quantum technology hinder its further progress. Specifically, the control qubit and target qubit of a two-qubit gate can only interact with adjacent specified qubit pairs.

**Figure 1:**
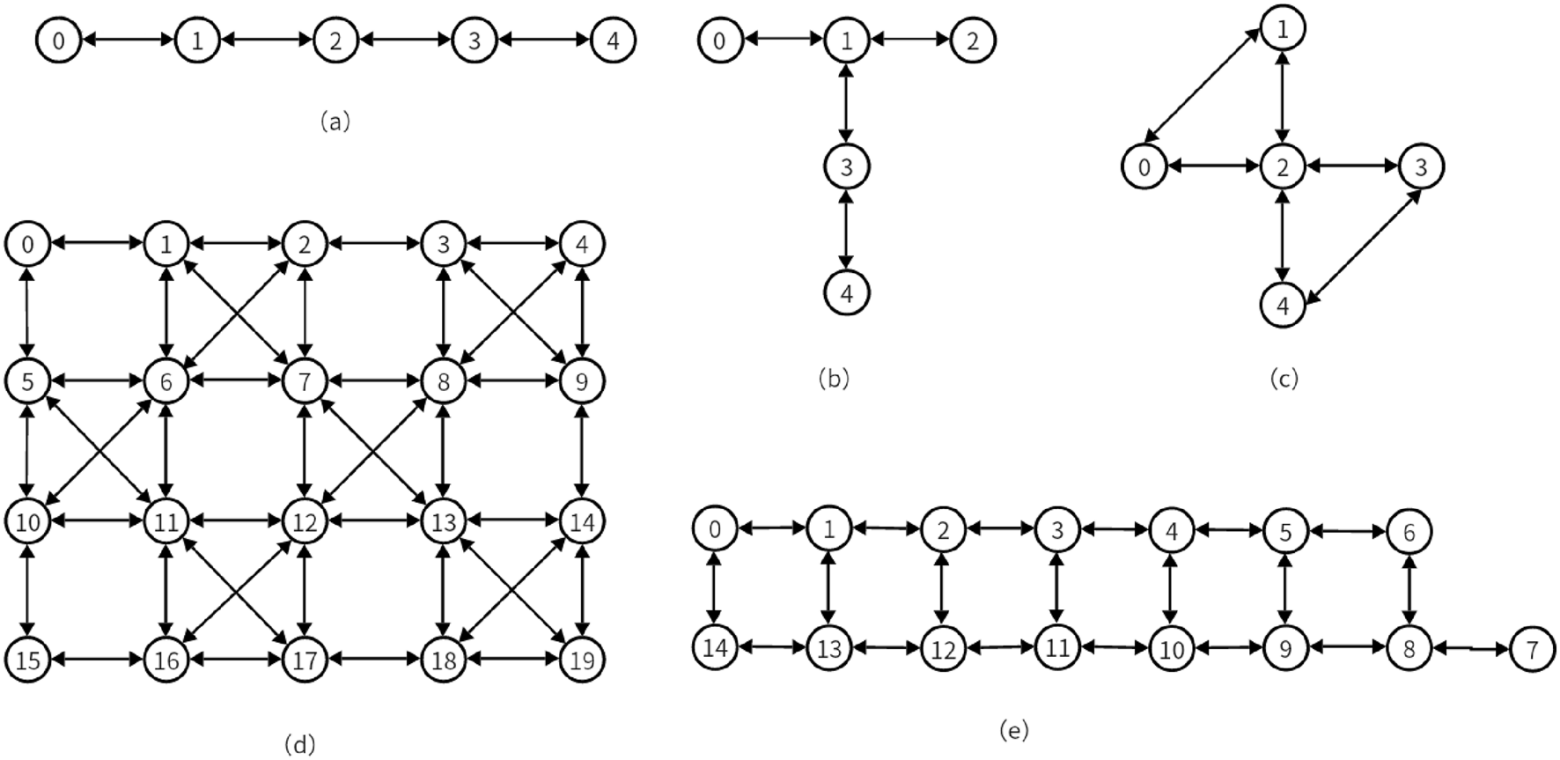
Qubit coupling diagram of IBM Q device. (**a**) IBM Q Santiago, (**b**) IBM Q Ourense, (**c**)Yorktown IBM QX2 v2.2.0, (**d**) IBM Q20 Tokyo, (**e**) IBM Q16 Melbourne.

Figure 1 shows the qubit topology of the quantum device, demonstrates various connecting ways between the coupled qubits^9^. As shown in the figure, all the qubits are placed on a planar geometry and bidirectional arrows are used to represent the connections between two qubits. Due to the limitations of the coupler, a physical qubit can only be connected to its neighboring physical qubit^10^. Usually, when designing quantum algorithms, the designers do not consider the limitations of the devices, allowing multiple-qubit gates to act on arbitrary qubits. However, a problem is ignored during this process. In the actual situation, quantum devices have their limitations regarding qubit interactions. It means that not all qubits can be connected, and different devices have unique topology diagrams for qubit connections.

In the past few years, intrigued by the qubit mapping problem, more and more scholars have devoted themselves to further its study^11,12^. When tackling it, qubit mapping requires considering a series of qubit gate operations in a circuit and enabling all double-qubit gates to comply with the coupling restrictions of a quantum device by inserting SWAP gates. However, the insertion of SWAP gates leads to an increase in quantum circuit depth, noise, and quantum computation time, inevitably jeopardizing the fidelity of the algorithm^13^. As a result, an efficient algorithm for mapping qubits in logic circuits to qubits in physical chips is urgently needed, to minimize the impact of side effects, like noise, and maintain the fidelities of quantum computation.

To address the qubit mapping problem, researchers have proposed various methods, which can be broadly categorized into two types in general. The first class of methods formulates the qubit mapping problem as an equivalent mathematical problem. Wille et al.^14^ use inference engines such as Boolean satisfiability to obtain the minimum solution, while Bhattacharjee et al.^15^ propose an integer linear programming formulation to achieve the minimum logical depth of a quantum circuit. In the second class of methods, heuristic algorithms are mainly adopted for problem-solving. Among these layers, a heuristic approach is used to further screen out the optimal SWAP gate insertion choices between layers during the mapping process. Li et al.^16^ propose a bi-directional heuristic search algorithm based on SWAP using a new backward traversal technique to optimize the initial mapping globally and introduce a decay effect to achieve a trade-off between depth and gate number for the whole algorithm. Besides, regarding the problem of finding a qubit mapping as a subgraph isomorphism problem, Matsuo et al.^17^ suggest an SAT-based method for finding an excellent initial mapping for quantum circuits, which is combined with a heuristic clustering algorithm can effectively reduce the number of inserted SWAP gates. Eesa Nikahd et al.^18^ propose an automated method called Window-based Quantum Circuit Partition, which aims to minimize the communication cost between processing units in distributed quantum computing.

Currently, most practical qubit-mapping methods for NISQ devices are based on heuristic algorithms^19^. Therefore, this paper focuses on heuristic-based qubit 10 allocation methods. Although the available heuristic algorithms usually solve the qubit mapping problem in an acceptable time, some limitations still leave adverse effects^20^. For example, in the literature^21^, it is stated that there is more room to improve the generation of the initial qubit mapping. A single division by distance is inappropriate, as it may lead to missing the optimal mapping in many cases. In the literature^13^, a prospective approach is adopted to evaluate the cost of SWAP insertion. However, the window size of the prospective technique is fixed, which means that without considering the internal details of the quantum circuits, the optimal SWAP operations would be missed in many cases.

Given all that, this paper proposes the dynamic and look-ahead-based qubit mapping method for more efficiently solving the qubit mapping problem caused by the connectivity constraints between the physical qubits of NISQ devices. More specifically, the proposed method is a qubit initial mapping algorithm based on the idea of maximizing physical bit connectivity, which generates a set of qubit initial mappings according to the input quantum circuit information and the selected topology map. The algorithm not only optimizes the expression of physical bit connectivity by preferentially selecting the physical qubits adjacent to the mapped logical qubits but also takes into account the interactions between qubits as well as the order in which the quantum gates are executed, thus generating a better initial qubit mapping.

However, due to the limitation of quantum hardware, the coupling relationship between physical bits is often limited, and the generated initial mapping can only satisfy part of the coupling relationship. For this reason, we propose a heuristic search algorithm based on SWAP, which employs a multi-window look-ahead strategy and takes the Minimal Subsequent Positive Effect (MSPE) of the SWAP operation as a heuristic cost function. This heuristic algorithm dynamically searches for the optimal SWAP insertion strategy. Compared with the IBM qubit mapping strategy, the method in this paper reduces the number of SWAP gates inserted on the IBM Q16 Melbourne by up to 30.36% and reduces the number of SWAP gates by 12.94% on average. Compared to the SABRE algorithm, the method in this paper inserts up to 23.95% fewer SWAP gates on IBM Q20 Tokyo, with an average of 14.2% fewer SWAP gates.

## Results

To better evaluate the effectiveness of the qubit mapping algorithm proposed in this paper, this algorithm is compared with the three methods integrated with the IBM Q Quantum Cloud platform, the SABRE^16^ algorithm, and the algorithms proposed in the literature^21^.The algorithms integrated with the IBM Q Quantum Cloud platform have been credited for its high credibility and reliability^12^, Sabre is a classical and representative algorithm, and^21^ is the basis of algorithm improvement in this paper. Therefore, they are selected as the baselines for the experiments in this paper.

### Comparison with IBM solutions

Table 1 lists the experimental results compared with the BasicSwap qubit-mapping strategy, which is the default mapping algorithm used in IBM Q. The first three columns are the information related to the input circuit, i.e., the name of the circuit (Name), the number of qubits (n), and the number of gates (G). The 5th and 6th columns indicate the number of basic gates of the hardware-compatible circuits obtained using the algorithm proposed in this paper and BasicSwap, respectively, and the 7th column indicates the difference between the 5th and 6th columns in percentage.

**Table 1:** Compared with IBM Q’s BasicSwap qubit-mapping strategy.

Name	n	G	Ours	IBM	Comp (%)
3_7_13	3	36	54	60	10.00
4_49_16	5	217	337	372	9.41
4gt4-v0_73	6	395	623	669	6.88
4gt4-v1_74	6	273	426	483	11.80
4gt5_77	5	131	194	225	13.78
4gt10-v1_81	5	148	235	267	11.99
4gt11_82	5	27	39	56	30.36
4gt11_84	5	18	27	36	25.00
4gt12-v0_86	6	251	398	410	2.93
4gt12-v1_89	6	228	357	394	9.39
4gt13_90	5	107	161	196	17.86
4gt13-v1_93	5	68	104	124	16.13
4mod5-bdd_287	7	70	112	130	13.85
4mod5-v0_18	5	69	111	124	10.48
4mod5-v1_23	5	69	117	124	5.65
4mod7-v0_94	5	162	246	283	13.07
4mod7-v1_96	5	164	248	271	8.49
aj-e11_165	5	151	235	276	14.86
alu-bdd_288	7	84	138	170	18.82
alu-v0_26	5	84	129	151	14.57
alu-v1_29	5	37	58	62	6.45
Average			207	232	12.94

As shown in Table 1, the algorithm in this paper reduces the total number of basic gates in the mapped circuit. In the BasicSwap algorithm, the initial mapping of logical qubits is generated sequentially, e.g., logical qubit q0 is assigned to physical qubit Q0, logical qubit q1 is assigned to physical qubit Q1, and so on. However, in most cases, there are better choices than such an assignment without negatively affecting the subsequent SWAP-gate insertion. Instead, the better initial mapping strategy for quantum circuits based on the IQM method proposed in the previous section reduces the number of subsequent SWAP gate insertions, alleviating the potential risk. Overall, the method in this paper reduces the number of SWAP gates by 12.94% on average and up to 30.36%.

In addition, other algorithms are integrated into IBM Q, such as LookaheadSwap and StochasticSwap, which can insert SWAP gates into the quantum circuits to make them compatible with the coupling mapping. To further the study, the qubit mapping algorithm proposed in this paper is compared with LookaheadSwap and StochasticSwap, with the experimental results presented in Table 2. The table’s first column is the name of the benchmark circuit, and the second column is the number of SWAP gates added by the algorithm in this paper. The third to fifth and sixth to eighth columns denote the number of SWAP gates added by the LookaheadSwap and StochasticSwap methods, respectively, for optimization levels ranging from 1 to 3. A higher optimization level means that the circuit is optimized at the cost of a longer execution time.

**Table 2:** Comparison with IBM’s LookaheadSwap and StochasticSwap strategies.

Name	Swap count						
	Ours	LookaheadSwap			StochasticSwap		
		L = 1	L = 2	L = 3	L = 1	L = 2	L = 3
3_7_13	6	9	8	8	10	10	9
4_49_16	40	109	104	69	66	67	62
4gt4-v0_73	76	176	201	97	127	115	120
4gt4-v1_74	51	113	133	77	90	87	85
4gt5_77	21	56	45	45	42	40	36
4gt10-v1_81	29	45	45	45	49	41	41
4gt11_82	4	6	6	6	13	10	8
4gt11_84	3	4	4	4	8	6	5
4gt12-v0_86	49	101	120	66	74	74	72
4gt12-v1_89	43	81	121	65	78	72	69
4gt13_90	18	36	44	40	34	33	31
4gt13-v1_93	12	25	20	20	20	20	20
4mod5-bdd_287	14	29	35	30	20	24	19
4mod5-v0_18	16	22	21	21	23	23	27
4mod5-v1_23	28	81	97	48	43	46	48
4mod7-v0_94	28	77	94	46	40	47	57
4mod7-v1_96	28	56	60	39	44	50	48
aj-e11_165	18	33	36	31	31	35	29
alu-bdd_288	15	25	30	21	22	26	32
alu-v0_26	7	9	10	10	10	10	9
alu-v1_29	6	9	8	8	10	10	9

The LookaheadSwap method takes a forward-looking approach to evaluate the impact of SWAP-gate insertion. However, the limit in this forward-looking approach is adopting a fixed-size window, which is challenging to satisfy all quantum circuits in the actual situation to optimize qubit mapping. In this paper, the size of the forward-looking window is flexible, and hence, it can find a better SWAP-gate insertion strategy. As shown in Table 2, the number of SWAPs added by the algorithm in this paper is smaller than that of the LookaheadSwap method, proving its efficiency. The StochasticSwap method maps qubit by inserting randomly selected SWAPs. However, the nature of this method cannot guarantee that an optimal solution can be obtained every run. As a result, through the comparisons, the method proposed in this paper outperforms the StochasticSwap method in terms of algorithmic stability and efficiency.

### Comparison with SABRE

In this section, experiments are carried out targeting the 20 qubit IBM Q20 Tokyo quantum processor, as shown in Fig. 1d, and compared with the SABRE algorithm proposed by Li et al.^16^. For a fair comparison, we utilized the publicly available SABRE code on the qiskit and compared it to our experiments, and the results of the experiments are shown in Tables 3 and 4.

**Table 3:** Experimental results of small-scale circuits.

Name	n	G	SABRE		Ours		Comp
			g1	t_1_ (s)	g_2_	t_2_ (s)	(g_1_ − g_2_)/g_1_ (%)
4mod5-v1_22	5	21	0	0	0	0.036	0.00
mod5mils_65	5	35	0	0	0	0.059	0.00
decod24-v2	4	34	0	0	0	0.043	0.00
4gt13_92	5	66	0	0	0	0.081	0.00
ising_model_10	10	480	0	0.004	0	0.313	0.00
rd84_142	15	343	196	0.012	192	0.565	2.06
AVERAGE							0.34

**Table 4:** Experimental results of medium-scale circuits.

Name	n	G	SABRE		Ours		Comp
			g_1_	t_1_(s)	g_2_	t_2_(s)	(g_1_ − g_2_)/g_1_ (%)
radd_250	13	3213	1275	1.98	1107	9.096	13.17
z4_268	11	3073	1365	2.93	1128	8.005	17.36
sym6_145	7	3888	1290	4.63	981	9.235	23.95
cycle10_2_110	12	6050	2622	11.58	2298	39.790	12.35
adr4_197	13	3439	1641	5.69	1272	11.830	22.48
misex1_241	15	4813	1521	8.21	1335	13.929	12.22
AVERAGE							16.92

According to the number of quantum gate operations, quantum circuits can be classified into different scales, such as small-scale, medium-scale, and large-scale circuits. The experimental results are shown in Tables 3, 4 and 5. The first column in the table is the name of the benchmark circuit, the second column is the number of qubits in the circuit, and the third column indicates the number of gates in the circuit. The fourth column, g_1_, labeled “SABRE”, shows the number of CNOT gates added by the SABRE algorithm, and the fifth column, t_1_, indicates the time required to run the SABRE algorithm. The sixth column, g_2_, labeled “Ours”, denotes the number of auxiliary CNOT gates added by the qubit mapping algorithm proposed in this paper, and the seventh column, t_2_, represents the time required to run the algorithm in this paper. The last column labeled “Comp.” indicates the gate number difference between our and the SABER algorithms.

**Table 5:** Experimental results of n large-scale circuit.

Name	n	G	SABRE		Ours		Comp
			g_1_	t_1_ (s)	g_2_	t_2_ (s)	(g_1_ − g_2_)/g_1_ (%)
co14_215	15	17,936	8982	39.82	3333	1765.96	62.89
square_root_7	15	7630	2598	17.05	1029	247.62	60.39
sym9_193	10	34,881	16,653	211.51	5743	2201.48	65.51
sqn_258	10	10,223	4344	35.02	1340	130.53	69.15
rd84_253	12	13658	6147	36.72	2380	390.40	61.28
9symml_195	11	34,881	14,790	131.81	5743	3000.36	51.73
cycle10_2_110	12	6050	2622	13.54	897	84.37	65.79
Average							62.40

As shown in Table 3, for small-scale circuits, the circuit gate number optimization effect of this paper’s algorithm and the SABRE algorithm are almost the same. As shown in Table 4, the optimization effect of this paper’s algorithm is more significant when it comes to the medium-sized circuits, with the highest optimization rate reaching 23.95%. The SABRE algorithm finds an initial mapping by iterative bi-directional routing, using the reverse traversal techniques. Next, it adopts a heuristic search scheme to reduce the number of swaps inserted during compiling the quantum program. However, the SABRE algorithm has a different initial mapping each time it is executed. We ran the algorithm 5 times and selected the best result among them. This paper uses the IQM algorithm to generate a better initial mapping and the forward-looking algorithm to insert SWAP gates efficiently without needing multiple runs.

Meanwhile, as seen from Tables 3 and 4, the algorithms proposed in this paper do not have an advantage regarding compilation time as it is not the research goal. We aim to reduce the number of gates because the noise and distortion introduced by gate operations are crucial challenges in quantum computation. Various errors such as decoherence, qubit flipping, or phase shifting may occur during the execution of gate operations, which cumulatively may lead to erroneous computational results. Reducing the number of gate operations can directly lead to less impact of noise and distortion in quantum computing systems. Hence, to improve the correctness and stability of computation, the algorithms proposed in this paper focus on the number of SWAP gates and sacrifices the compilation time performance to a certain extent.

### Comparison with QCM

After comparing with the QCM algorithm in the literature^21^, the experimental results are acquired and shown in Table 6. In this table, the first column is the name of the reference circuit, and the second column is the number of qubits in the circuit. The third column indicates the number of gates in the circuit, while the fourth column, labeled “QCM”, g1, indicates the number of CNOT gates added by the QCM algorithm. The fifth column, t_1_, represents the time required to run the QCM algorithm. The sixth column, g_2_, labeled “Ours”, denotes the number of auxiliary CNOT gates added by the qubit mapping algorithm proposed herein, and the seventh column, t_2_, is for the time required to run the algorithm herein. The eighth and ninth columns labeled “Comp.” represent the number of gates and the compilation time compared between the QCM algorithm and ours.

**Table 6:** Comparison with QCM algorithm.

Name	n	G	QCM		Ours		Comp	
			g_1_	t_1_	g_2_	t_2_	(g_1_ − g_2_)/g_1_ (%)	(t_1_ − t_2_)/t_1_ (%)
3_7_13	3	36	54	0.163	54	0.065	0.00	60.40
4_49_16	5	217	358	0.17	337	0.240	5.87	− 41.05
4gt4-v0_73	6	395	647	0.298	623	0.353	3.71	− 18.42
4gt5_77	5	131	203	0.275	194	0.242	4.43	11.96
4gt10-v1_81	5	148	247	0.175	235	0.108	4.86	38.52
4gt11_82	5	27	32	0.139	39	0.134	− 21.88	3.28
4gt11_84	5	18	32	0.108	27	0.025	15.63	76.82
4gt12-v0_86	6	251	404	0.119	398	0.017	1.49	85.88
4gt12-v1_89	6	228	381	0.347	357	0.296	6.30	14.64
4gt13_90	5	107	173	0.23	161	0.257	6.94	− 11.83
4gt13-v1_93	5	68	104	0.125	104	0.097	0.00	22.17
4mod5-bdd_287	7	70	112	0.117	112	0.063	0.00	46.43
4mod5-v0_18	5	69	114	0.481	111	0.066	2.63	86.32
4mod5-v1_23	5	69	120	0.26	117	0.054	2.50	79.17
4mod7-v0_94	5	162	261	0.163	246	0.065	5.75	59.91
4mod7-v1_96	5	164	260	0.176	248	0.158	4.62	10.41
aj-e11_165	5	151	247	0.15	235	0.176	4.86	− 17.33
alu-bdd_288	7	84	141	0.168	138	0.130	2.13	22.57
alu-v1_29	5	37	58	0.121	58	0.065	0.00	46.57
Average							3.77	30.34

## Discussion

In this work, we proposed several methods to tackle the qubit-mapping problems. As for the qubit mapping initialization problem, this paper uses the connectivity of physical qubits and the priority of qubits to generate a relatively optimal initial qubit mapping, which avoids subsequent SWAP-gate insertions as much as possible. In addition, the proposed method mentioned of inserting SWAP gates is proven to be effective for the qubit-mapping position-update problem. The method, based on the idea of multi-window look-ahead dynamically, inserts SWAP gates efficiently. The proposed algorithms can alleviate the coupling limitation of quantum devices and reduce the cost of mapping quantum circuits to NISQ devices. It has been shown that our work can alleviate the coupling limitation of quantum devices and has more flexible prospective depth, resulting in cost reductions for qubit mapping through the diminished insertion of SWAP gates.

However, there are other factors that can affect the execution of quantum computing, such as quantum gate execution error and quantum bit coherence time. These factors can be taken into consideration in future research. Additionally, arranging and optimizing quantum gates can reduce the number of quantum gates, which not only lowers the complexity of the computation but also enhances the fidelity of the operations. This is an important direction for optimization in quantum circuit design^22^. Our future research direction will be to adjust the algorithms proposed in this paper to account for these factors.

## Methods

**Figure 2:**
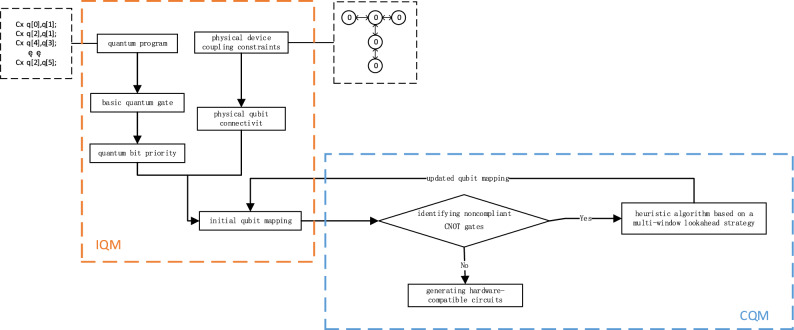
Qubit mapping algorithm flowchart.

As for the problem that quantum hardware only allows double-qubit gates to act between a limited number of neighboring physical bits, a qubit-mapping algorithm is proposed in this research. As shown in Fig. 2, the algorithm is mainly divided into two parts: initialization of qubit mapping (IQM) and change of qubit mapping (CQM). IQM is a mapping strategy that generates the initial qubits, while CQM selects the best SWAP-gate insertion based on the initial mapping generated by IQM to change the qubit mapping.

In the IQM phase, the quantum gates in the quantum procedure are first decomposed into basic quantum gates that can be directly applied to the NISQ device, and the qubit priority is determined based on the number of qubits acted on by the CNOT gate. Then, based on the topology of the selected quantum device, the connectivity of the physical qubits of the device is obtained. Finally, an initial qubit mapping is generated based on the obtained qubit priority and the connectivity of the physical bits.

In the CQM stage, the CNOT gates in the quantum program are first traversed to find gates that do not meet the coupling constraints according to the initial qubit mapping strategy that has been generated. Then, a multi-window look-ahead heuristic algorithm is taken to insert SWAP gates to update the qubit mapping, and the process is looped until the end of the CNOT gate traversal.

### Initialization of qubit mapping (IQM)

To further improve the effectiveness of the initial mapping algorithm, we can introduce another metric called qubit interaction. The qubit interaction takes into account the execution order of quantum gates and the interaction relationships between qubits in the quantum circuit.

#### Qubit interaction

A physical quantum device can be represented by its coupling graph CG, which is an undirected graph (V, E) where each qubit in the device is a node in V, and there is an edge (q_i_, q_j_) 2 E between two nodes q_i_ and q_j_ if they can be operated by a two-qubit gate in the device.

##### Definition 1

Given a quantum circuit LC = (Q, C), where Q = {q_0_, q_1_,…, q_n-1_} be a set of logical qubits, and the quantum circuit C = {g_0_, g_1_, ..., g_m-1_} is a set of ordered gates. Assign a weight w_i_ to each qubit gate g_i_, and the w_i_ is

1 $${w}_{i}=m-i.$$

##### Definition 2

For each qubit pair $({q}_{i}\text{,}{\text{q}}_{j})$, where ${q}_{i}\text{,}{ \, {\text{q}}}_{j}$ are qubits in LC, the weight ${\text{QPI}}({q}_{i}\text{,}{\text{q}}_{j})$ is

2 $${\text{QPI}}\left({q}_{i}\text{,}{\text{q}}_{j}\right)=\sum_{{g}_{i}}{w}_{i},{ g}_{i}=\left({q}_{i}\text{,}{\text{q}}_{j}\right)or\left({\text{q}}_{j}\text{,}{q}_{i}\right),$$

where w_i_ is the weight of g_i._

##### Definition 3

Let assign q_i_ to Q_j_, and the qubit interaction is

3 $$ {\text{QBN}}\left( {Q_{i} } \right) = \mathop \sum \limits_{i,j = 0}^{{{\text{count}}}} {\text{QPI}}\left( {M\left( {Q_{i} } \right),M\left( {Q_{j} } \right)} \right),i \ne j,Q_{i} {,}Q_{j} \mathrel\backepsilon A,{\text{Dist}}\left[ {Q_{i} } \right]\left[ {Q_{j} } \right] = 1, $$

where M(Q) refers to the logical qubit corresponding to the physical qubit Q, A denotes the set of physical qubits that have been assigned, count denotes the number of physical qubits that have been assigned, and ${\text{Dist}}[{Q}_{i}][{Q}_{j}]=1$ denotes that Q_i_ is adjacent to Q_j_.

The weight (w_i_) of the quantum gate (g_i_) indicate the sequence of quantum gates, where a higher number suggests that the quantum gate is placed earlier in the sequence. The ${\text{QPI}}({q}_{i}\text{,}{ \, {\text{q}}}_{j})$ refers to the total number of the weight of a pair of qubits interact in a circuit.

##### Example 1

**Figure 3:**
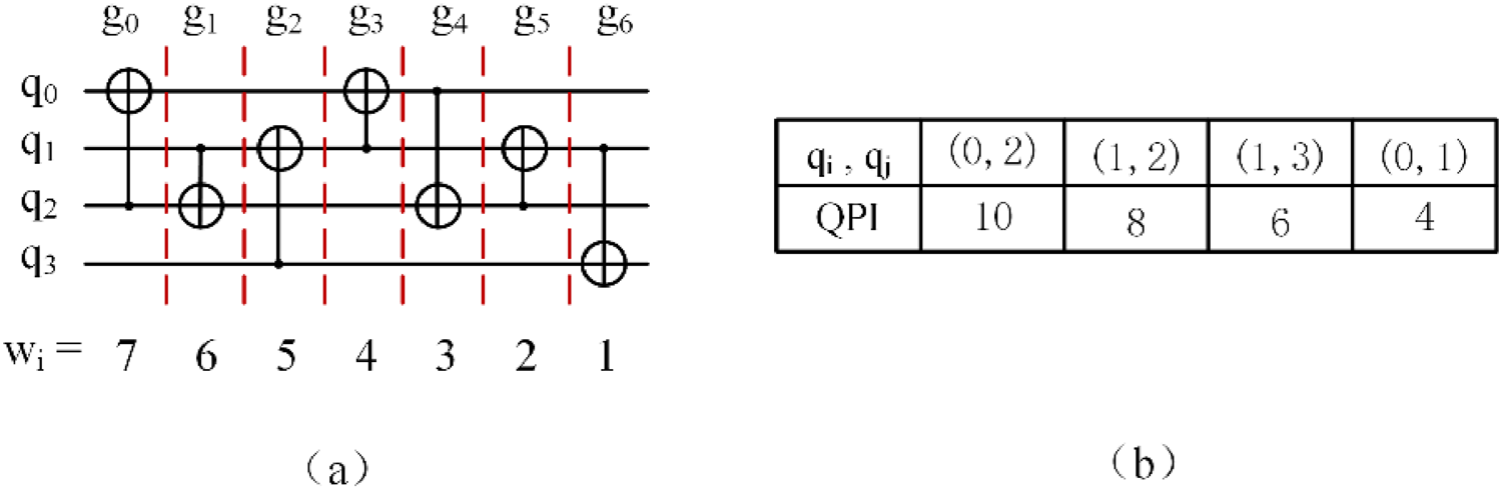
(**a**) The weight of each gate in the circuit, (**b**) the QPI of each qubit pair.

Figure 3 displays the weights of the gates in the circuit and the QPI of each qubit pair. The QPI (q_0_, q_2_) has the highest weight indicates that the gates associated with these qubits are executed first and more frequently in the circuit.

#### Initial qubit mapping

##### Priority of logical qubits

The logical qubit priority is determined by the number of two-qubit gates applied on that qubit in the quantum program. If two qubits have the same priority value, a higher priority is given to the qubit that appears earlier in the circuit.

##### Physical connectivity strength (PCS)

In this paper, the definition of the PCS of a physical qubit is the sum of the number of its first-neighboring qubits and the sum of its second-neighboring qubits. A qubit’s second neighboring qubit refers to a first-neighboring qubit of one of its first-neighboring qubits but not itself or its first-neighboring qubit. The levels of neighboring qubits included in PCS relate to the scalability. For architectures with more qubits, it may be appropriate to include higher-level neighboring qubits in PCS, such as third-neighboring qubits and fourth-neighboring bits.

The PCS of a physical qubit is the number of its neighboring physical qubits. Therefore, when a physical qubit has a larger PCS, the logical qubit mapped to that physical qubit would have a more significant chance of connecting to other logical qubits, resulting in a low probability of moving. Conversely, when it comes to a physical qubit with a low PCS, fewer neighboring physical qubits are around it, indicating that more movements would be needed to satisfy the coupling constraints. These movements would require more SWAP-gate insertion, causing more possible errors. Therefore, the core idea of generating the initial mapping in this paper is to place the logical qubits with more interactions at the physical qubits with larger PCSs.

Algorithm 1 outlines the steps for constructing an initial mapping, where the input of the algorithm is the list of logical qubits sorted in descending order of QPI and the set PCS of all the physical bits, and the output of the algorithm is the quantum mapping M. More detailed, the algorithm is designed in the following way. First, the qubit with the highest QPI is assigned to the physical qubit with the highest PCS, the most connected physical qubit. Then, before the next logical qubit assignment, whether any of its logical neighbors have been placed should be checked. If none have been placed, the unassigned physical qubit with the highest PCS would be selected for allocation. Otherwise, among the unallocated physical neighbors of placed qubits, the physical qubit with the highest QBN value would be chosen. If multiple scenarios exist for the maximum QBN value, all mapping scenarios will be saved.

**Algorithm 1:**
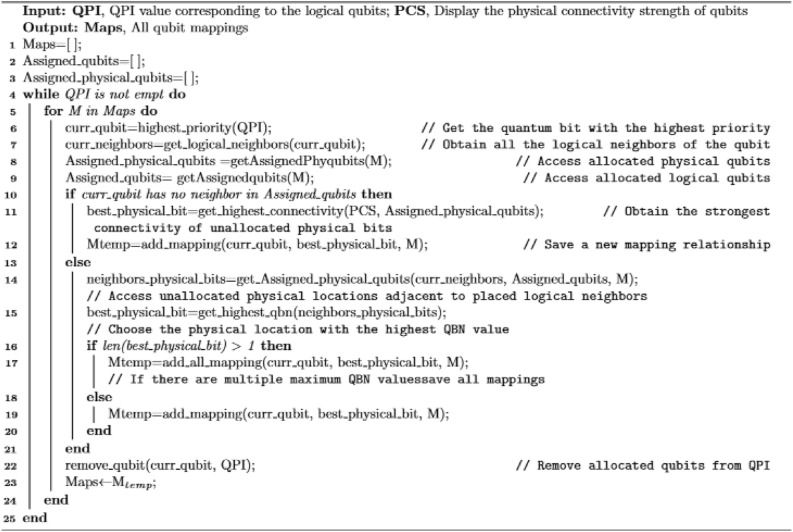
IQM.

###### Example 2

**Figure 4:**
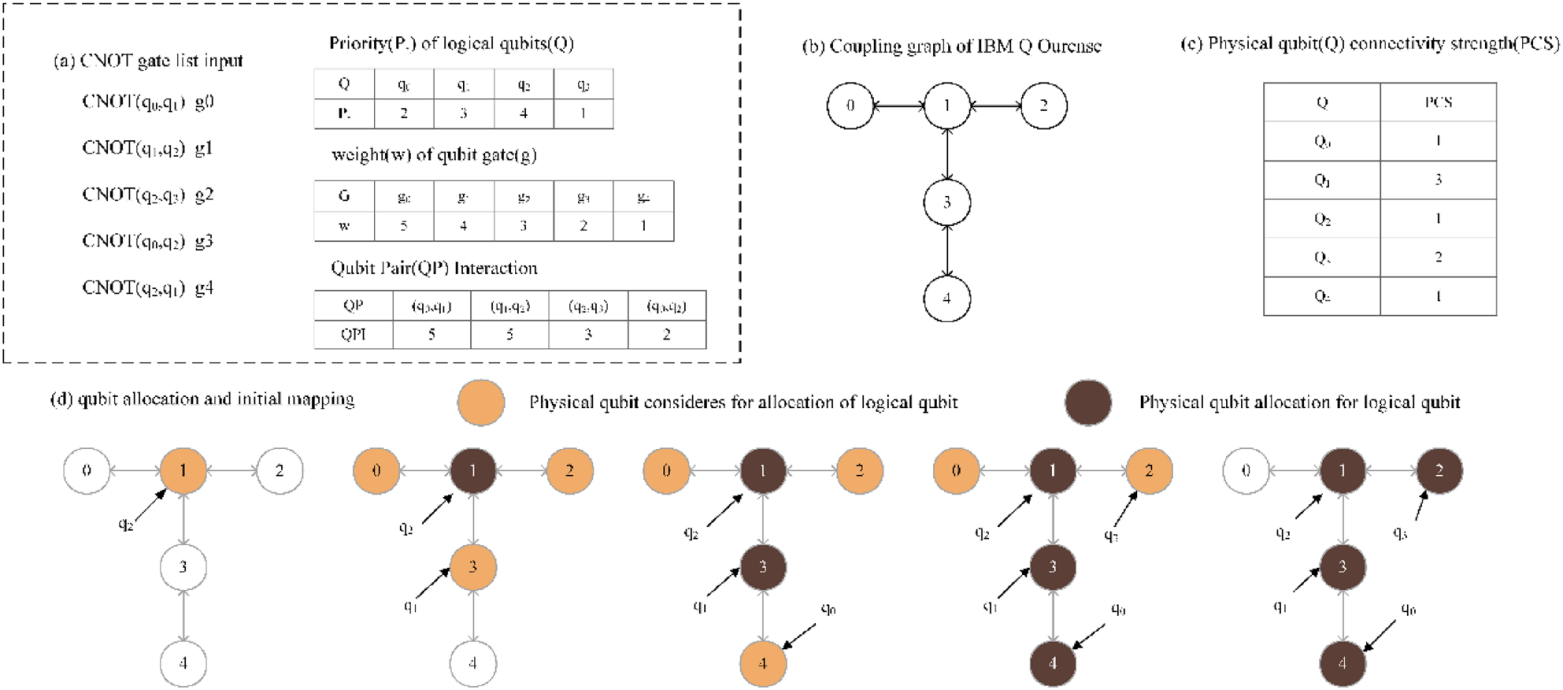
(**a**) Coupling graph of a 5-qubit quantum computer from IBM (ibm q ourense), (**b**) connectivity strength metrics of different qubits in ibm q ourense, (**c**) a original circuit with Priority of logical qubits and Qubit Pair Interaction, (**d**) qubit allocation and initial mapping.

The initial mapping construction process of the quantum circuit LC in Fig. 4a and the coupling diagram CG in Fig. 4b is shown in Fig. 4c,d. The logical qubit allocation sequence is shown in Fig. 4a, which is q_2_, q_1_, q_0_, q_3_. In this example, the PCS of a physical qubit is the sum of the number of its first-neighboring qubits. The complete hardware profile of ibm q ourense is shown in Fig. 3c.

First, logical qubit ‘q_2_’ has a physical qubit candidate Q_1_, directly assigned to Q_1_. Then, Logical qubit ‘q1’ has 3 possible andidates (as it is a logical neighbor of ‘q_2_’), all have same QBN each and Q_3_ is picked randomly in the example. And Logical qubit ‘q_0_’ has 3 possible andidates, and Q_4_ has the highest QBN. Therefore, Q_4_ is chosen for ‘q_0_’. The other qubits ‘q3’ are placed to Q_2_ respectively in a similar fashion.

### Change of qubit mapping (CQM)

After generating the initial bit mapping, some violated constraints in the quantum circuit must be resolved further. The essential problem is that the connections between physical qubits in a quantum device are finite. When mapping a double-qubit gate to a limited number of physical qubit pairs, SWAP-gate insertion is needed to change the qubit mapping to accommodate the coupling constraints of the physical device. Inserting SWAP gates at different positions has different impacts on subsequent quantum gates. Therefore, to better measure this impact, this paper proposes a heuristic algorithm based on multi-window look-ahead for calculating the impact of inserting SWAP gates on the operation of subsequent gates.

Specifically, regarding the Minimal Subsequent Positive Effect (MSPE) of the SWAP operation as a heuristic cost function, this algorithm calculates the effect of each insertion of a SWAP gate on the subsequent double-qubit gates, providing the best insertion operation for the local part. The MSPE is expressed as follows shown in the following:

4 $${\text{MSPE}}=\Delta \text{d}+{\sum }_{i}^{i+w}Dist\left({g}_{i}\right).$$

**Figure 5:**
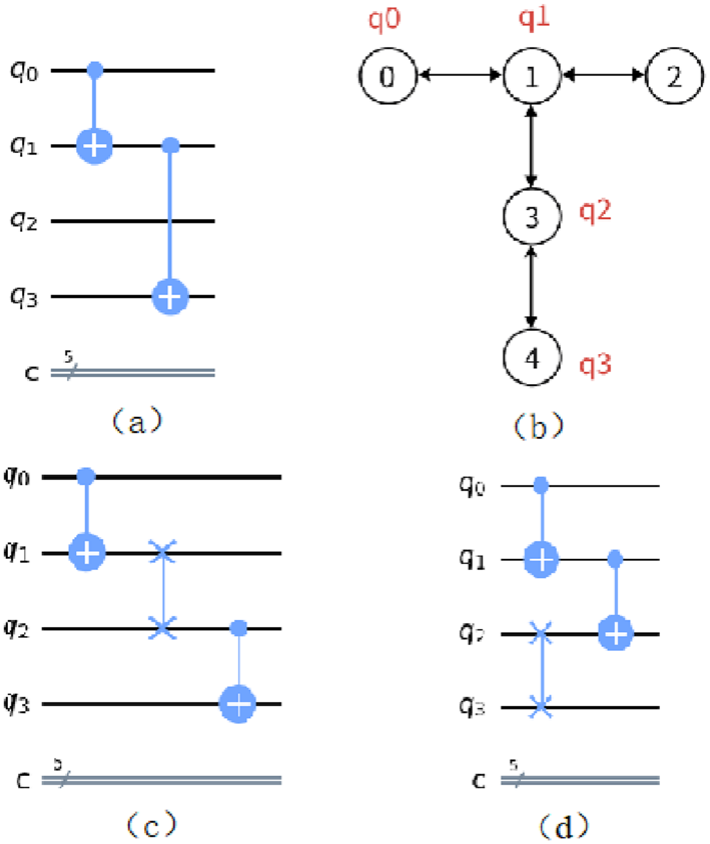
Circuit $\Delta \text{d}$ analysis chart when different SWAPs are used (**a**) Original Quantum Circuit, (**b**) Physical Qubit Coupling Graph Example, (**c**) Insert one SWAP operation between q1 and q2, (**d**) Insert one SWAP operation between q2 and q3.

In this equation, G_i_ refers to the CNOT gate that does not match the mapping, and w is the window size, indicating the number of gates to be considered afterward, starting from the first gate. The window size grows until the optimal solution is obtained. Supposing the case that more than one optimal solution is obtained, the window size is increased by 1, and the MSPE value is recalculated until a minimum solution is obtained or the window reaches the tail of the circuit. $\Delta \text{d}$ represents the impact of the SWAP gate on increasing the circuit depth. The quantum circuit in Fig. 5a is mapped like Fig. 5b, and then SWAP gates must be inserted. Calculated by Eq. (4), the $\Delta \text{d}$ value is different for both insertion methods in Fig. 5c,d. However, the $\Delta \text{d}$ of the circuit in Fig. 5c is zero, and the $\Delta \text{d}$ of the circuit in Fig. 5d is one. Finally, the flow of the SWAP insertion is demonstrated in “Methods” section.

**Figure 6:**
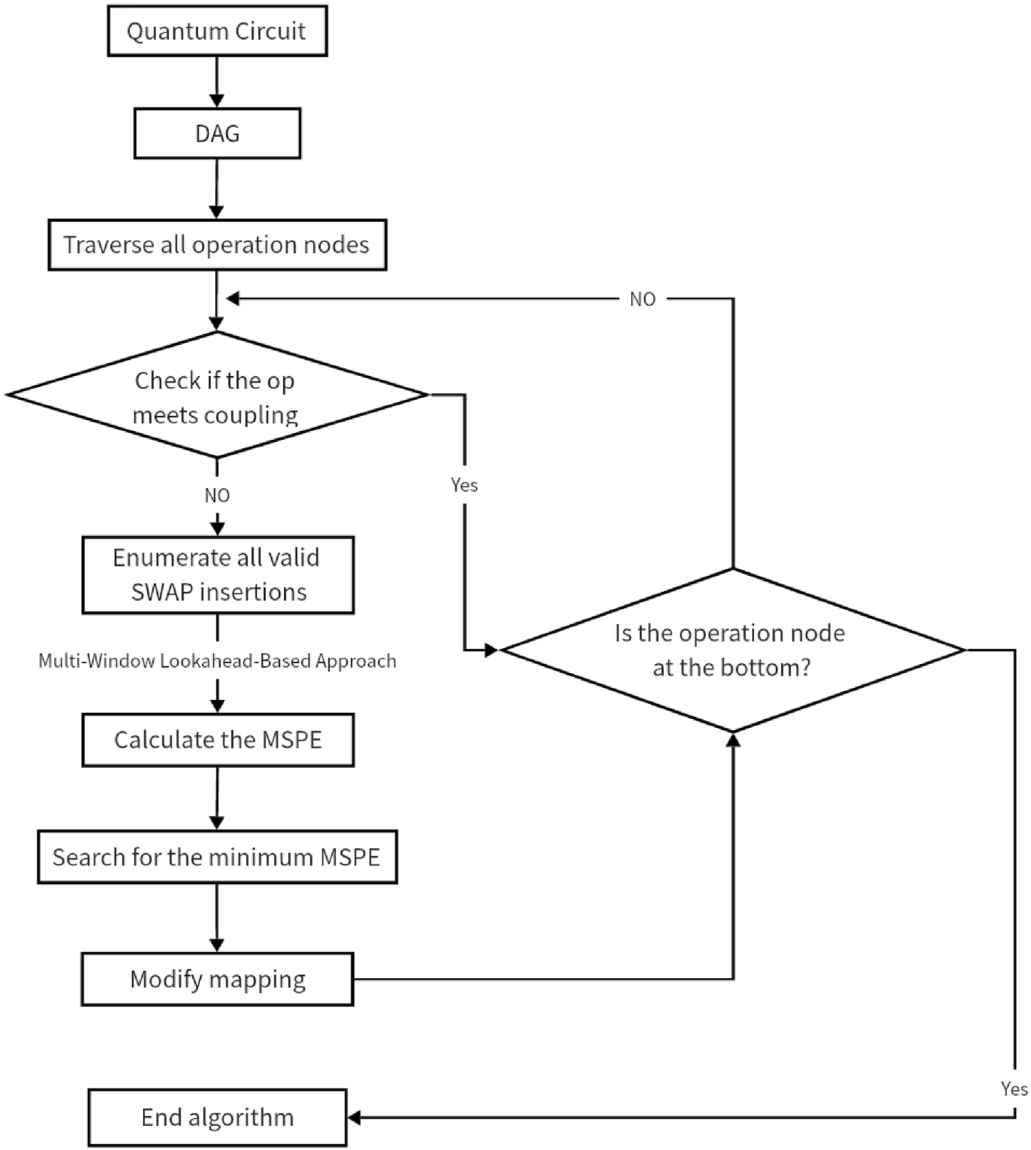
Flow chart of CQM algorithm.

The flow of the CQM algorithm is demonstrated in Fig. 6. More detailed, in the first step, the input quantum circuits are converted into a DAG, and all double-qubit gates in the DAG are traversed layer by layer. Next, according to the current mapping, check whether the traversed double-qubit gates are consistent with the coupling diagram of the quantum device. If they are inconsistent, it is necessary to enumerate all the effective SWAP-gate insertions. Finally, the most effective SWAP-gate insertions are selected based on the multi-windowed forward-looking algorithm. The pseudo-code of CQM is shown in Algorithm 2.

**Algorithm 2:**
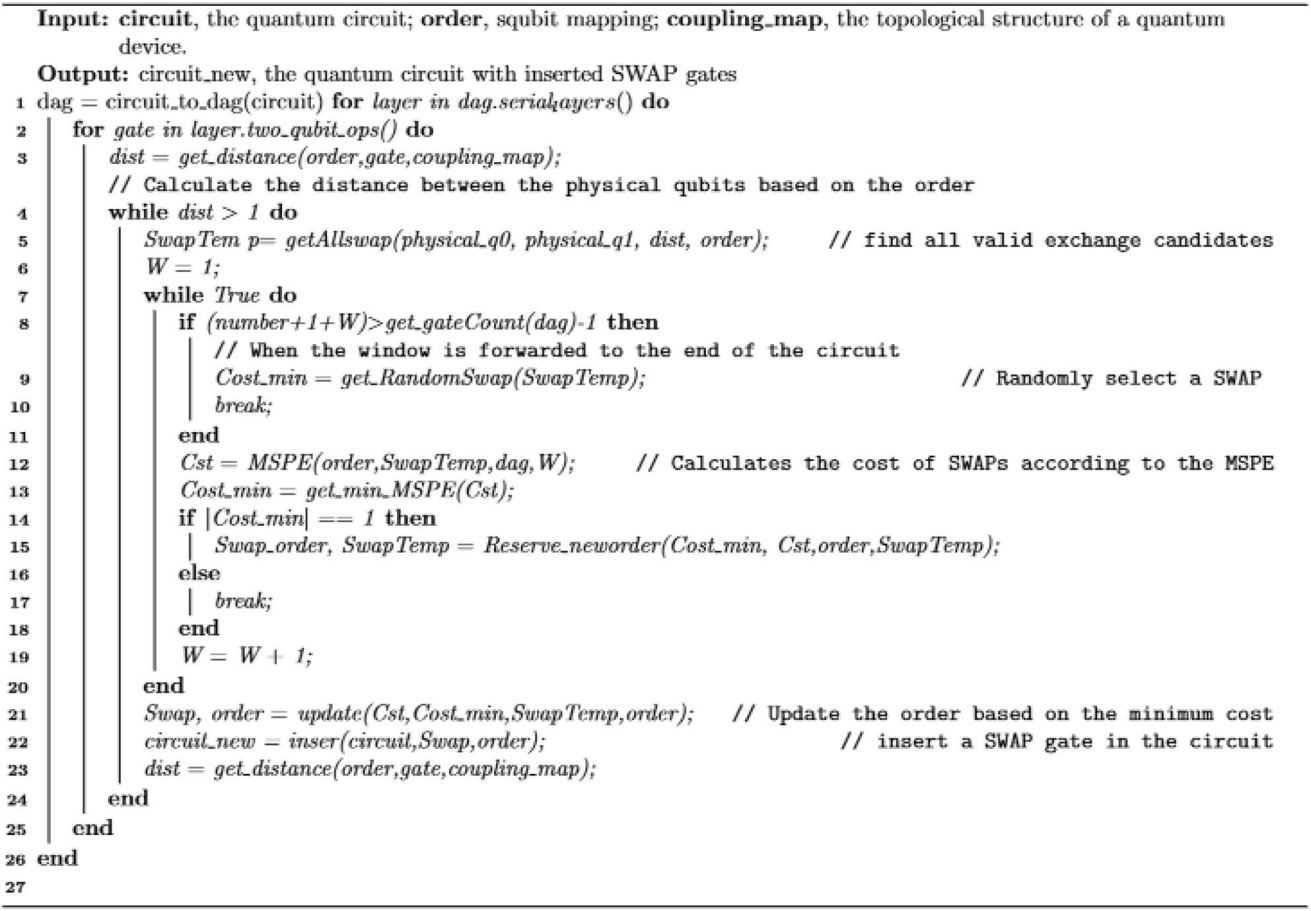
CQM.

For the implementation details and source code, please refer to our GitHub repository: https://github.com/xxxxzbj/QM-DLA.

## Data Availability

The authors declare that all data supporting the findings of this study are available within the article. Source data and codes can be accessed via proper request from the corresponding author. Competing interests The authors declare no competing interests.
